# Healthcare professionals’ experiences with the use of continuous glucose monitoring in nursing homes and home healthcare services: a qualitative study

**DOI:** 10.1186/s12877-026-07414-w

**Published:** 2026-03-31

**Authors:** Anne Merete Vik Aslestad, Anne Haugstvedt, Monica Hermann

**Affiliations:** 1 Western Norway University of Applied Sciences, Department of Health and Caring Sciences, Bergen, Norway; 2 Western Norway University of Applied Sciences, Department of Health and Caring Sciences, Klingenbergveien 14, Stord, N-5414 Norway

**Keywords:** Diabetes, Insulin, Glucose monitoring, Continuous glucose monitoring, CGM, Qualitative study, Nursing home, Healthcare worker

## Abstract

**Background:**

Continuous glucose monitoring (CGM) is commonly used to monitor glucose levels in younger people with type 1 diabetes, but its use is less frequent among older adults. The aim of this study was to investigate the experiences of healthcare workers who have used CGM in the follow-up of glucose levels in older people with type 1 or type 2 diabetes living in nursing homes or receiving home healthcare services.

**Methods:**

The study had a qualitative design, and four focus group interviews with a total of 16 healthcare workers were conducted. The interviews were analysed using Braun and Clarke’s thematic analysis.

**Results:**

Through the analysis four themes with two or three subthemes each, were identified. The themes were (1) **“**CGM affects the workday and provides an overview” (subthemes “Detects hypoglycaemia”, “CGM with tracking function would be resource-saving” and “Varied experience with alarm function and trend arrows”); (2) “Practical training is important, but not sufficient” (subthemes: “Theoretical diabetes knowledge is a prerequisite for the full utilization of CGM” and “Access to a diabetes nurse is perceived as valuable and reassuring”); (3) “Improved dialogue and increased ability to participate in activities” (subthemes: “Avoids painful pricks which disrupts the dialogue”, “Increased focus on the patient” and “Less equipment to carry” and (4) “Changed roles and distribution of responsibilities” (subthemes: “Increased understanding of glucose values creates a sense of security” and “More independent patients and more reassured relatives”.

**Conclusions:**

This study revealed that CGM positively affected the workday of healthcare workers, the follow-up of patients, and the use of resources. The participants experienced that the use of CGM improved the dialogue between healthcare workers and patients and contributed to patients becoming more independent. However, increased knowledge about diabetes and CGM among healthcare workers is crucial to optimize the utilization of the CGM technology.

## Background

The prevalence of diabetes increases with increasing age, and according to the International Diabetes Federation, the highest prevalence is in the age group 65–79 years [1]. Diabetes places significant demands on self-management, which can be challenging for older people with declining physical and cognitive health. A significant proportion of individuals with diabetes rely on insulin therapy to manage their condition, and the number of home-dwelling people aged 65 and older using insulin in Norway has more than doubled over the last two decades [2, 3]. This includes people with both type 1 and type 2 diabetes. Using insulin increases the risk of hypoglycaemia, which is an adverse effect of insulin [4].

The treatment and follow-up of diabetes need to be personalized, and the goal is to avoid emergencies caused by hypoglycaemia and prevent complications caused by hyperglycaemia [5, 6]. For older adults, the primary guideline recommendation is to minimize hypoglycaemic events [7]. Nevertheless, several studies have shown that unrecognized hypoglycaemia is common in older people with diabetes [8–11]. This is both due to age-related changes impairing the ability to recognize symptoms of hypoglycaemia, as well as unspecific symptoms of hypoglycaemia, such as unsteadiness, falls or nausea [12, 13]. The American Diabetes Association recommends that older people with diabetes using insulin perform capillary blood glucose measurements to prevent hypoglycaemia [7]. For many home-dwelling older people with diabetes, this is challenging due to comorbidities that reduce their ability to perform self-management tasks, such as glucose monitoring and insulin injections, thereby placing the responsibility on their family. If the need for assistance increases, home healthcare services, or ultimately nursing home placement, are the alternatives [14]. A study from France showed that around 50% of insulin-treated individuals 75 years or older with type 2 diabetes handled insulin themselves or got help from relatives, while the rest needed support from healthcare workers [9]. In Norway, 15–20% of those who need help from home healthcare services have diabetes [15].

A continuous glucose monitoring (CGM) device consists of a small sensor sitting just under the patients’ skin and is used to measure interstitial glucose throughout the entire day. The user can access historical glucose data and get a round-the-clock capture of the glucose levels. The information is possible to share with others through a tracking function on an application. In addition, the user can turn on an alarm function which notifies when the glucose level either rises or falls. The use of CGM is rising and in Norway approximately 90% of people with type 1 diabetes use CGM [16]. Mayberry et al. has reported an increased use of CGM also in type 2 diabetes [17]. The technology is, however, less used among older people with diabetes, although studies indicate that CGM can be useful for achieving adequate glucose control in individuals with type 2 diabetes of all ages [18–21].

Although a recent study indicated that the use of CGM in older people with type 1 diabetes is cost-effective, costs have been highlighted as a barrier for the use of CGM in older people [22]. Also, need for staff training, frequent alarms, and the possible removal of sensors by patients with cognitive impairment have been used as arguments against the use of CGM [23]. Although older people have the same rights to health and care services as younger people, CGM is still rarely used among older adults with diabetes receiving home care services or nursing home residents, in Norway. Also, research on the use of CGM in community health care services, is limited. Therefore, the use of CGM in the follow-up of older people with insulin-treated diabetes was tested in community health care services in a rural municipality in Southern Norway. The aim of this study was to investigate the experiences of healthcare workers with the use of CGM in the patients 65 years or older with insulin-treated diabetes in nursing homes and home health care services.

## Methods

### Study design

To explore the healthcare workers experiences with the use of CGM, we used a qualitative approach. The COREQ checklist for qualitative studies was used [24].

### Setting and participants

The study was conducted in a rural municipality in Southern Norway, where the use of CGM was tested in the follow-up of older people with diabetes who used insulin and received community health care services. We recruited healthcare workers (registered nurses, auxiliary nurses and assistants) that were working in home healthcare services or nursing home. An auxiliary nurse has a shorter education and less responsibility compared with a registered nurse. The organization of the municipality’s service is divided in three zones, whereof one zone has a nursing home and the two others have assisted living facilities. The staff working in home care services provides service in both the assisted living facilities and in people’s homes, while nursing home personnel work only in the nursing home. The inclusion criterion was to have experience with the use of CGM in patients aged 65 and above with diabetes in home care or nursing homes. The work in the municipality is organized without dedicated roles for CGM tasks; all registered nurses, auxiliary nurses and assistants with extra training in medication management may be involved in follow-up of individuals using CGM. Therefore, all 183 individuals working as nurses, auxiliary nurses or assistants in the municipality received an invitation by e-mail to participate in the study. Only 9 individuals responded to this invitation. Subsequently, everybody with a permanent position in one zone (*n* = 20) were contacted by telephone, of which 7 accepted the invitation. In total, 16 healthcare workers, all women between 22 and 65 years old, agreed and provided written consent to participate in this interview study (Table 1). The participants worked as registered nurses, auxiliary nurses or assistants. Their work experience in the current position ranged from 2 months to 33 years.

**Table 1:** Overview of the participants and demographic data

Interview	Participant	Age (years)	Work experience (years)	Work title	Workplace
1	1	> 50	> 10	Registered nurse	Nursing home
1	2	> 50	> 10	Auxiliary nurse	Nursing home
1	3	< 35	1–5	Assistant	Nursing home
2	1	< 35	< 1	Registered nurse	Home care
2	2	35–50	6–10	Registered nurse	Nursing home
2	3	> 50	6–10	Registered nurse	Home care
2	4	35–50	1–5	Auxiliary nurse	Nursing home
3	1	< 35	1–5	Registered nurse	Home care
3	2	35–50	> 10	Registered nurse	Home care
4	1	> 50	> 10	Auxiliary nurse	Nursing home
4	2	> 50	> 10	Auxiliary nurse	Nursing home and home care
4	3	> 50	1–5	Auxiliary nurse	Nursing home and home care
4	4	< 35	< 1	Registered nurse	Nursing home
4	5	< 35	1–5	Auxiliary nurse	Home care
4	6	< 35	< 1	Registered nurse	Nursing home
4	7	35–50	6–10	Auxiliary nurse	Nursing home

### Data collection

Semi-structured focus group interviews were used for data collection. This was chosen because the use of focus groups allows exploration and discussion of complex themes among the participants, which can potentially result in more profound expressions of the individuals’ opinions [25].

The interviews took place in the participants work time at their workplace. No financial compensation was provided. Four focus groups with two, three, four, and seven informants were conducted. The interviews lasted from 26 to 48 min and were audio-recorded. An interview guide, developed by the three authors, was used during the interviews (Table 2). The interview guide included follow-up questions to ensure that all relevant topics were covered. If the conversation between the participants stopped, the interviewer used follow-up questions as support. The first author, AMVA, conducted all four interviews, and they were performed at the participants’ workplaces. All interviews were transcribed verbatim by AMVA. Field notes were recorded during the interviews to capture non-verbal communication. The transcripts were not returned to the participants for comments or corrections.

**Table 2:** Overview of the key questions and sub-questions in the interview guide

Key questions	Possible sub-questions
Can you tell me about your involvement in monitoring older adults with CGM?	What responsibilities have you undertaken in relation to the follow-up process? How is the distribution of responsibilities? Who does what (patients, relatives, staff)? Has the use of CGM affected your workday and resource utilization in any way? Can you explain further?
Based on your experiences, what do you think about the use of CGM for elderly individuals with type 1 and type 2 diabetes who receive municipal health care services?	Advantages and disadvantages? Can you give examples? Is there a need for training of healthcare personnel to manage this? What effect have the trend arrows had?
What is your experience with the alarm function on CGM?	Are the older individuals able to manage the alarms themselves? How do you receive notifications about alarms? What are the procedures for following up on alarms? Are the alarms being documented?
Can you tell me about any challenges you have experienced related to the use of CGM?	Technical difficulties? Patients who express discomfort or dissatisfaction? Measurements that you cannot rely on? Sensor detaches too early, rash, or pain? How is it managed? What are your thoughts on the responsibilities related to CGM, glucose monitoring, and insulin management?
Do you experience any difference in the follow-up of individuals with diabetes - those with CGM versus those without?	Are there any specific challenges for those with CGM? Are there any specific challenges for those without CGM? (finger pricking) How does CGM affect the collaboration between you and the patient and/or next of kin? Are there any reasons why elderly individuals do not wish to try CGM? What are the differences before and after the use of CGM?
Closing question	
Is there anything we haven’t covered that you would like to say more about?	

### Data analysis

Braun and Clarke’s reflexive thematic analysis was used to analyse the transcribed interviews. Thematic analysis offers tools for developing, analysing, and interpreting patterns within a qualitative dataset [26]. It involves a systematic process of coding the data to identify and create themes. All authors collaborated on the analysis. AMVA is an experienced diabetes nurse, AH is both an experienced diabetes nurse and diabetes researcher while MH is an experienced diabetes researcher. Additionally, AH has many years of lived experience with type 1 diabetes.

The analysis was performed through a recursive process within the six phases of thematic analysis, which are described in Table 3 [26]. NVivo15 was used to code and manage data [27]. In accordance with the six phases, the authors first read and re-read all data items separately to become familiar with the dataset. Separately, we also recorded initial ideas and generated initial codes (phase 1–2). In the next phase, all authors met for a workshop for discussion of the initial codes and organised them into potential themes (phase 3). Further in workshops, the themes were reviewed and re-organized several times (phase 4). In this process, new codes and themes were created for data that did not fit into the previous coding phase. Finally, we agreed on the extracted themes and meaningful patterns and subthemes (phase 5).

**Table 3:** The six phases of thematic analysis according to Braun and Clarke [26] and description of the analytical process

Phases	Description of the process
1. Get familiarized with the data	Reading and re-reading of transcripts. Documenting initial ideas.
2. Generate initial codes	Coding interesting features of the data systematically across the entire dataset. Collating data relevant to each code
3. Generate initial themes	Developing initial themes from the codes by clustering potentially connected codes.
4. Develop and review themes	Further developing and reviewing the viability of candidate themes from phase 3 through a process of re-engagement with all the coded data and the entire dataset. Checking that all themes address the research question and tell a convincing story of the data
5. Refine, define and name themes	Refinement of each theme and the overall story. Deciding on an informative name of each theme.
6. Produce the report	Writing the analytical report and contextualizing the analysis in relation to existing literature.

### Ethics

The project was approved by the Regional Committee for Medical and Health Research Ethics (2018/512/REK) and Norwegian Agency for Shared Services in Education and Research (302932/SIKT). All participants signed written consent forms and were informed of their right to withdraw from the study at any time. We ensured the anonymity of the participants and patients by using no names and removed identifying features in the transcripts. The audio files were deleted when the analysis was finished.

## Results

From the analysis of the focus groups interviews, we identified four themes, and each theme comprised two or three subthemes regarding healthcare workers’ experience with CGM. The themes and subthemes are presented in Table 4.

**Table 4:** Overview of themes and subthemes generated by the analysis of focus group interviews (*n* = 4) with healthcare workers on their experience with continuous glucose monitoring in older adults with diabetes living in nursing homes or receiving home healthcare services

Themes	Subthemes
CGM affects the workday and provides an overview	Detects hypoglycaemia
	CGM with tracking function would be resource-saving
	Varied experience with alarm function and trend arrows
Practical training is important, but not sufficient	Theoretical diabetes knowledge is a prerequisite for the full utilization of CGM
	Access to a diabetes nurse is perceived as valuable and reassuring
Improved dialogue and increased ability to participate in activities	Avoids painful pricks which disrupt the dialogue
	Increased focus on the patient
	Less equipment to carry
Changed roles and distribution of responsibilities	Increased understanding of glucose values creates a sense of security
	More independent patients and more reassured relatives

### CGM affects the workday and provides an overview

The participants said that CGM in various ways affected their workday as healthcare workers. They pointed out that this technology offered several advantages, such as detecting hypoglycaemia, reviewing glucose history, and helped them in the planning of follow-up. CGM was also experienced to be time-efficient and the healthcare workers reported feeling more secure. One participant described that these advantages resulted in more stable glucose levels:

*“I think you can keep a much more stable glucose level… since you*,* you automatically measure more often and then you can adjust [insulin doses] … better*,* I think. So*,* I have no doubt that there are great advantages.”* (Interview 4, participant 2).

Some of the participating healthcare workers used CGM to review the history of glucose levels and received a comprehensive overview throughout the entire day. In that way CGM helped them to detect hypoglycaemic episodes that would not have been identified with capillary measurements. When they detected hypoglycaemia, the insulin doses were changed, and the glucose levels got more stable. One participant who used the glucose history said:

*“And once we had them (CGM) and were able to use them*,* we incorporated them*,* which allowed us to identify hypoglycaemia in the morning.”* (Interview 2, Participant 3).

The participants said they spent less time monitoring glucose levels after the patients received CGM, compared to measuring capillary blood glucose. In addition, the threshold was lower for conducting glucose-control at night, as they didn’t have to wake up the patient. They just needed to use the CGM, and they would have the glucose level in seconds. This was helpful in situations where they otherwise would have been uncomfortable leaving the patient’s home because they were afraid that a hypoglycaemic event could happen. The fear of hypoglycaemia often led healthcare workers to provide additional supervision. One participant described it like this:

*“It’s just that it’s much more reassuring with that sensor (CGM)*,* because then you can check several times if you get scared that the glucose level… is going down or… and it makes it easier for us to… To keep an eye on things. When you don’t have the ability to be there all the time.”* (Interview 4, participant 4).

The participants discussed the tracking function of the CGM, which enabled them to receive notifications in the event of hypoglycaemia. The informants said it made them more secure and that it could save resources by avoiding the need for additional supervision. One of the healthcare workers in the home healthcare services described it like this:

*“If the night shift could have the patient’s glucose levels on a phone*,* it would help… that they could monitor the patients throughout the night. They could avoid having to physically visit*,* as they could just check the measurements on their phone.”* (Interview 3, participant 2).

The informants expressed that it was difficult to trust new technology for themselves and for some patients. This resulted in varied use of the alarm function and trend arrows. The healthcare workers had experienced patients that became stressed by the alarms, and therefore the alarm function was turned off. However, they described individual differences between the patients, and that some of them used the alarm function to receive alerts for low glucose levels. The informants had experienced some confusion by the trend arrows among the healthcare workers. In addition, they were a bit unsecure because CGM in fact measures the interstitial glucose rather than the blood glucose. They mentioned that there was a delay in glucose values when measuring interstitial glucose. One of the informants got confused with the trend arrow, even when there was no reason for it. She described it like this:

*“… it [CGM] shows that the glucose is 6*,* and the arrow is straight across. Then I am like ….[gasp]*,* but actually… logically speaking*,* when you measure it the old way [capillary] at 6*,* then you are completely calm.”* (Interview 1, participant 1).

Healthcare workers with more experience using CGM described how they utilized trend arrows and glucose history to determine whether it was urgent to administer insulin, adjust the dose, or advise the patient to eat. The CGM made it easier to plan further follow-up.

### Practical training is important, but not sufficient

The participants described the use of CGM to control the glucose levels before and after meals in the same way as they did with capillary measurements. Also, they described the need for education to manage and utilize the possibilities with CGM. The informants experienced that connecting to a CGM was quite easy and that it was easy to check the glucose level. However, they felt that there were several functions on the CGM that some healthcare workers did not have enough knowledge to utilize. In addition, the CGM provided information that the healthcare workers needed more education and knowledge about in order to utilize and avoid uncertainty. One participant described it as this:

*“We could perhaps have had more education on how to use it to its full potential.”* (Interview 3, Participant 2).

The informants said it was an advantage that the CGM was easy to use for checking the glucose level. They compared it to the capillary glucose measurements that also requires education to avoid errors. They emphasized that there are several sources of error with capillary measurements that they did not have to think about with CGM. This made it easier to instruct temporary workers, who had varying levels of competence. One healthcare worker described the challenge:

*“We do have temporary workers*,* of course. And even though we try to instruct everyone*,* it may happen that not all of them have equal competence in… how to perform capillary measurements correctly… so you might not always get accurate results. That can be a challenge.”* (Interview 3, Participant 1).

The participating healthcare workers had access to a diabetes nurse working in the municipality. The diabetes nurse had advanced knowledge about CGM in diabetes management. The informants highlighted that access to such expertise provided security and was reassuring. They were uncertain if the responsible physician had the same knowledge about CGM, thereby they preferred that the diabetes nurse assisted them with decisions based on CGM data.

*“It definitely feels safer for us - that there is a diabetes nurse who follows them up*,* rather than a responsible physician.”* (Interview 1, participant 1).

### Improved dialogue and increased ability to participate in activities

The informants highlighted that not all patients understood the necessity of glucose monitoring. When they used capillary glucose measurement, the healthcare workers often spent time persuading patients to control the glucose. The participants expressed a feeling that the patients got tired of the capillary measurements. The patients’ fingertips became sore, and they didn’t want the healthcare workers to conduct the glucose measurement. They also felt that they were causing the patient discomfort by pricking their fingers all the time. One informant described the procedure as a disruption to the dialogue with the patient.

*“So*,* you… get a much better dialogue with a patient who has a CGM*,* because then*,* then you don’t have to bother them.”* (Interview 4, participant 2).

The healthcare workers experienced that using CGM instead of capillary measurement helped them to focus on the patient rather than putting effort into encouraging the patient to accept a capillary glucose control. They described situations with capillary measurement as time-consuming and difficult because the focus shifted towards controlling glucose rather than focusing on the patient. This was especially the case when they had to perform an ad hoc control of the glucose level. In situations like that, one informant felt the patient’s lost confidence in her. She described it like this:

*“I almost feel sometimes that the patients can become like they lose confidence in me*,* or like they go “oh*,* again*,*” just because you are uncertain*,* you know.”* (Interview 4, participant 4).

The informants described that some patients experienced less need for help from healthcare workers when they used CGM. It required less time to control glucose, and it was less noticeable to other people in the same room when a glucose control was performed. In addition, there was less equipment to carry everywhere, which made it easier for patients to participate in activities.

### Changed roles and distribution of responsibilities

The informants described that when using CGM, some patients became more independent. They emphasized that checking glucose levels with CGM was more manageable for the patients.

*“Patients whom we had to help with measuring their glucose*,* and now they can do it themselves.”* (Interview 1, participant 1).

The informants described how the information from CGM enhanced their understanding of glucose values. They perceived that this applied to the patients, as well. They recognized that some patients’ awareness of the glucose fluctuations led them to be more careful with what and how much they ate of food that caused high glucose levels. As a result, some patients experienced more stable glucose levels.

*“He [the patient] is good at using the CGM and sees that the glucose level rises*,* and that it is actually carbohydrates that cause it [glucose level] to rise.”* (Interview 2, participant 3).

In addition, the participants expressed that those patients that used the alarm function responded to alarms and ate food when experiencing low glucose or they called for assistance. One participating healthcare worker talked about a patient that didn’t take responsibility, but after he got CGM this changed. She described it like this:

*“…but I think it seems like they also become more independent… he [the patient] feels he has a bit better control.”* (Interview 2, participant 1).

The participating healthcare workers described that some family members of the patients had expressed some advantages with the use of CGM, for example that the use of CGM made it easier for them to take the patient home. Although some issues with the CGM could happen, where they had to call the home care service for help, the overall experience was that it provided more reassured relatives and that the use of CGM was a wish from several relatives.

*“It’s a wish from the relatives’ side too*,* because they feel safer… that their… family member… would be more closely monitored if they had a CGM.”* (Interview 4, participant 4).

## Discussion

In this study we interviewed healthcare workers employed in nursing homes and home healthcare services who had experience with the use of CGM in older adults. We identified that CGM positively affected their workday by offering reassurance through an overview of glucose levels and detecting hypoglycaemia. Additionally, CGM gave the healthcare workers more time for dialogue with the patients without the disturbance of finger pricks, contributed to more efficient use of healthcare resources and supported the patients’ independence. However, the results indicate a need for increased knowledge about diabetes and CGM among healthcare workers to fully utilize the technology.

CGM enables detection of blood glucose fluctuations, and in line with several previous studies our findings indicates that CGM contributes to detecting hypoglycaemia [8–10]. Older people have an increased risk of nocturnal hypoglycaemia and it is shown in several studies that it is often underdiagnosed [9, 10, 28]. The participants in our study emphasized their ability to detect hypoglycaemia early in the morning, by using the history of glucose levels from the CGM. Moreover, the participants said it was easier for healthcare workers to monitor glucose levels at night when patients had CGM. This is consistent with a previous study where family members reported that CGM made it easier to control glucose levels at night, without waking up the person with diabetes [29]. The informants expressed a difference in the follow-up of older patients with CGM versus those without. They noted that the tracking function of the CGM could contribute to resource savings by reducing the need for continuous supervision of patients.

Throughout the interviews, it became clear that some patients turned off the alarm function on the CGM to avoid confusion. This is in contrast to Litchman et al., who found that older adults perceive the alarms useful because they get an alert if the glucose level either rises or falls and that this helps reduce concerns about hypoglycaemia [30]. Several previous studies have shown that the majority of older adults are positive towards the alarms, although a few people find them annoying [29–31]. Furthermore, if the alarm is turned off it can result in hypoglycaemia because the older patient does not recognize the symptoms. Educating patients about the use and features of CGM, including the value of alarms, is essential to ensure the patient’s confidence in the reliability and functions of the systems and to make full use of it. This has also been pointed out in previous research, and the need for patient education has been identified as an essential barrier to the use of CGM [32, 33]. Similarly, lack of knowledge and training among healthcare personnel is also a key barrier to the implementation and use of CGM in both community and hospital settings [23, 33–36]. This was also pointed out by the healthcare workers in our study who emphasized the need for education on using trend arrows, tracking, and alarm function, regardless of their varying levels of experience with CGM features. However, the healthcare workers with more experience with CGM expressed that they had greater use of its various functions, which enhanced their ability to fully leverage the technology.

A holistic approach to diabetes care that includes both the physical and mental aspects of living with diabetes, in addition to helping people to strengthen their skills and autonomy for diabetes self-management is crucial and recommended within diabetes care [37]. The healthcare workers said CGM positivity affected the dialogue with patients, allowing them to have a more holistic approach to patient care rather than persuading patients to check their glucose levels using capillary measurement. Improved communication between healthcare professionals and patients was also found in a study examining the perspectives of nurses on the use of CGM in haemodialysis patients with insulin-treated diabetes [38]. As Huang et al. pointed out, managing glucose levels with capillary measurements can be challenging in older individuals [39]. In accordance with our study, Laursen et al. also emphasized that it took less time to control the glucose level, and that the patients became more engaged in their diabetes management when using CGM [38]. The participants in our study reported that some patients became more independent after they received CGM, as it enabled them to control their glucose levels themselves, and consequently reduced the need for assistance from healthcare workers and promoted patient autonomy. This is in alignment with the findings of Polonsky et al., who described less use of healthcare services when using CGM [40].

The participants said it was easier for patients to visit their family when using CGM, because it was easier to control glucose levels, and it involved less equipment to take along. This is in line with previous findings, where carers reported great ease in monitoring glucose levels with CGM [29]. In addition, our study found that the technology made it easier for patients to participate in activities and that relatives expressed a sense of reassurance when their family members had a CGM. In home healthcare services, the healthcare workers are minimally present with the patient, and it is the patient or their relatives who manage the disease most of the time [41]. Thus, the perspective of the family members is particularly important in home care service and it is essential for healthcare workers to maintain communication with both the patient and their next of kins.

In this study, the use of CGM was shown to have several advantages. There is an ongoing discussion about the use of CGM in several countries, which includes issues related to cost, resources, the potential annoyance of alarm functions, and whether there is sufficient knowledge about this technology. To reduces cost, there have been discussions about whether intermittent use could be an alternative. However, this might be challenging for a patient group that may have cognitive impairments. In our study the participants perceived CGM as resource saving but emphasized that there is a need for more knowledge about this technology among healthcare workers.

### Strengths and limitations

The main limitation of this study was recruitment challenges, which led to two of the interviews consisting of only two and three participants, respectively. Such small group sizes reduce the range of perspectives, limit participant interaction, and increase the likelihood that the moderator’s influence will shape the discussion more strongly than in larger groups. We observed that participants were generally favourable towards CGM and reported few challenges, although being explicitly asked about this. This apparent consensus may have been facilitated by the small group format, but it could also reflect that several participants worked at the same nursing home and therefore had similar experiences. It is also possible that individuals with more critical views of CGM chose not to participate. Only a small proportion specified reasons for declining; these were exclusively practical constraints or a perception that they lacked sufficient familiarity with CGM to contribute meaningfully to a focus group. Healthcare workers declining to participate due to what they consider is lack of knowledge has probably led to a group of participants that are savvier than the average healthcare worker. This might explain why we did not observe any resistance to change, as has previously been pointed out as a major barrier for implementation of CGM in primary care management for patient with type 2 diabetes on insulin therapy [33].

The small group size and the clustering of participants from the same workplace is a limitation to the transferability of the findings. Nevertheless, in Norway both registered nurses, auxiliary nurses and nursing assistants are involved in the care of patients with diabetes, and the inclusion of multiple professional groups and settings (home health care and nursing homes) strengthens the transferability of the results. It is also worth noting that, while healthcare systems are structured differently across countries, the management of patients using CGM is largely similar. Therefore, it is likely that healthcare professionals in both Norway and other countries can benefit from the insights derived from our study.

## Conclusions

This qualitative study has provided an understanding of healthcare workers’ experience with CGM in older people with diabetes who receive home care services or reside in nursing homes. The results showed that CGM positively affected the follow-up of patients, use of resources and that use of the technology improved the dialogue between healthcare workers and patients and contributed to patients becoming more independent. The participants stated that they did not have sufficient knowledge to fully utilize the CGM, which indicates a need for increased knowledge about diabetes and CGM among healthcare workers. It can be assumed that with long-term use, the user of the technology will become more confident, and trend arrows, tracking function and the alarm function may be beneficial for both patients and healthcare workers.

## Data Availability

Data can be obtained from the first author upon reasonable request.

## Abbreviation

CGM: continuous glucose monitoring
